# Traditional and Electronic Cigarette Usage Patterns, Dependence, and Perceptions Among Ajman University Students

**DOI:** 10.3390/ijerph23020143

**Published:** 2026-01-23

**Authors:** Khaldoun Tabbah, Safielrahman Haitham Sami Elawaddlly, Ahmad Jalal Kanawati, Mahmoud Tariq Al Ammour, Abdulrahman Salem Abufanas, Dena Nashaat Hamza, Abdul Ilah Ghazwan Dakak, Doha Farouk Abdelhafiz, Mohamad Mohamad Munzer Madarati

**Affiliations:** 1 Clinical & Medical Education Department, College of Medicine, Ajman University, Ajman P.O. Box 346, United Arab Emirates; 2 Center of Medical and Bio-Allied Health Sciences Research, Ajman University, Ajman P.O. Box 346, United Arab Emirates; 3 College of Medicine, Ajman University, Ajman P.O. Box 346, United Arab Emirates; 202010539@ajmanuni.ac.ae (S.H.S.E.); 202010440@ajmanuni.ac.ae (A.J.K.);

**Keywords:** nicotine dependence, E-cigarettes, cigarettes, university students, MENA region, harm perception, dual use, shisha, public health

## Abstract

**Highlights:**

**Public health relevance—How does this work relate to a public health issue?**

- Nicotine use among university students is an emerging public health issue in the MENA region.
- This study evaluates cigarette and e-cigarette use patterns, dependence, and perceptions in young adults.

**Public health significance—Why is this work of significance to public health?**

- E-cigarettes were the most commonly used nicotine product among students.
- Dual use and early initiation were associated with higher dependence levels.

**Public health implications—What are the key implications or messages for practitioners, policy makers, and/or researchers in public health?**

- Youth-focused cessation strategies should be prioritized within university health programs.
- The stronger regulation of e-cigarette marketing and sales is needed to reduce youth uptake.

**Abstract:**

Background: Nicotine use among the youth has been on the rise, especially with the introduction of E-cigarettes. This has sparked concerns regarding E-cigarettes and traditional cigarettes in terms of patterns, dependence, and perceptions within the youth population, which are issues this study aimed to investigate. Methods: A cross-sectional survey was conducted among university students at Ajman University, which is in the Middle East and North Africa (MENA) region. Using the Cigarette Dependence Scale (CDS-12) and Penn State Electronic Cigarette Dependence Index (PS-ECDI), dependence on both cigarettes and E-cigarettes was quantified. Results: Out of 1713 respondents, 18.9% were currently using nicotine products, including E-cigarettes (12.7%) and traditional cigarettes (5.1%). Nicotine use was significantly associated more with males than females with an odds ratio of 4.14. However, there was no difference between genders in the dependence scores. In addition, dual nicotine use and an earlier onset of nicotine consumption were associated with significantly higher dependence scores than single users and a late onset of smoking. Participants overall attributed cigarettes and E-cigarettes as equally harmful. Conclusions: Both cigarette and e-cigarette use were prevalent and associated with notable dependence. Although E-cigarettes are often promoted as cessation aids, their use in our sample did not appear to facilitate quitting and may instead sustain nicotine dependence. Targeted youth-focused cessation programs and stricter marketing and sales regulations are essential to prevent further normalization. Longitudinal studies are needed to track evolving patterns and health impacts in the MENA region.

## 1. Introduction

Throughout the years, nicotine smoking has always been a major public health concern. It achieved public attraction with its numerous advertising campaigns, widespread availability, and increased popularity in the media. Moreover, industrialization allowed for extensively greater production rates, permitting easier access internationally [1]. Nicotine comes in many different forms including, but not limited to, cigarettes, E-cigarettes (vapes), shisha (waterpipe), and midwakh.

The consumption of tobacco products is shaped by social and cultural determinants such as peer influence, familial smoking, societal norms, and religious prohibitions, particularly in conservative regions like Saudi Arabia where smoking is stigmatized, especially among women [2]. Traditional cigarettes, which used to be the most commonly used form of smoking product, remain highly prevalent in today’s world. According to the World Health Organization (Global Health Observatory), the prevalence of current tobacco smoking worldwide is estimated to be around 18 percent, which corresponds to almost one in five individuals [3].

In the Middle East and North Africa (MENA) region, smoking prevalence varies, with some countries reporting higher prevalence among university students. Egypt (46.7%), Kuwait (46%), and Saudi Arabia (42.3%) were reported to have a high prevalence of smoking among university students by Nasser et al. [4]. In contrast, the United Arab Emirates shows a comparatively lower prevalence, although estimates vary by the population studied. While student-based data reported an overall prevalence of 15.1% [4], a large population-based study by Razzak et al. demonstrated smoking rates of 24.3% among males and 0.8% among females in the general adult population, reflecting a marked gender disparity [5].

When it comes to adverse effects, cigarettes are known for significantly worsening health and causing long-term conditions, such as cardiovascular and respiratory diseases and cancers [6]. Through atherosclerosis and vascular damage, cigarette smoking gives rise to coronary heart disease, cerebral vascular disease, and peripheral artery disease [7]. Moreover, chronic obstructive pulmonary disease, chronic bronchitis, and emphysema are commonly known lung conditions associated with smoking [8]. Lastly, cigarette smoking has been identified as a risk factor for around 12 types of cancers by the CDC, most notably lung cancer [9].

For this reason, E-cigarettes were marketed as safer options or cessation aids when they were introduced to the market, which led the population to greatly underestimate the harm that these devices could cause [10]. Adolescents and young adults, in particular, were targeted with advertisements about the safety and alluring flavors of vapes [11]. This has resulted in higher e-cigarette use amongst the younger population, with global prevalence estimates reaching 23% [12]. However, in the MENA region, the number of users among the general population is merely 2.2%, according to Jirjees et al. [13]. Conversely, the percentage of vapers was higher in a study performed in the UAE by Abbasi et al., where they found that 23% of university students had recently vaped [14].

Many studies have discussed the harm and dependence reduction aspect of E-cigarettes use, and they have found that although their usage decreases the percentage of cigarettes smoked per day, the “puff duration” of vapes is increased, which signifies an increase in dependence on them [15]. To understand why there was such a significant increase in usage among the youth, multiple papers proposed flavors, advertisement, and puff duration as factors that prolong dependency and therefore harm [15,16,17]. Ou et al. found that E-cigarettes were used for relaxation purposes and were also greatly desired due to their good taste [18]. Another study by Yang et al. showed that engagement with both pro- and anti-tobacco information at baseline predicted the subsequent onset of symptoms of dependence on E-cigarette products one year later [19]. Interestingly, Henn et al. mentioned that student vaping increased due to COVID-19 lockdowns, leading to an outcome of stress relief along with growing addiction rates [20].

The long-term effects of E-cigarettes are still unknown, but evidence of their toxicity and harmful effects has been emerging with each study [16]. E-Cigarette/Vaping-Associated Lung Injury (EVALI) is a novel life-threatening condition initially identified in 2019. Vitamin E acetate (VEA) and Tetrahydrocannabinol (THC), two compounds found in vapes, were noted as risk factors and possible causes of EVALI. This condition can present with shortness of breath, cough, chest pain, diarrhea, vomiting, fever, headache, dizziness, and, in some cases, hemoptysis. Eventually, it can progress to respiratory failure [21]. So far, the mainstay of treatment is only supportive with oxygen supplementation via non-invasive ventilation or mechanical ventilation if needed [22,23].

This paper examined E-cigarette and traditional cigarette use, dependence, and perceptions among university students at Ajman University. It also explored the association between cigarettes and e-cigarette dependence. Although our study is limited to one university, Ajman University is ranked the highest in diversity according to the 2026 QS World University Rankings [24]. By limiting this study to one university, it allowed for consistent and controlled data collection, ensuring uniform exposure to similar social and academic environments with the same university policy regarding nicotine use. While this limits generalizability, it strengthens the associations studied by increasing this study’s internal validity. This study offers valuable insight into E-cigarettes use and its relationship to traditional cigarettes among the youth.

## 2. Materials and Methods

### 2.1. Study Design and Population

A cross-sectional survey study was conducted at Ajman University across all colleges. The study period was from 17 September 2023 to 30 November 2024 (around 1 year and 2 months). The inclusion criteria included any Ajman University student who was enrolled during the period of this study, while the exclusion criteria included all Ajman University faculty and staff.

### 2.2. Study Sample

According to the university’s registration office, the number of enrolled students was 7113 for the duration of this study. Using Cochran’s sample size formula corrected for a finite population, a sample size of 365 participants was calculated. A Z-score of 1.96 with a 0.05 margin of error and an expected proportion of 0.5 was used for the calculation. A convenience sampling method was employed, in which responses were collected by invitations through student emails and in-person university events. All 7113 students were invited to participate in the survey. The final sample size was 1713 students (24.1% response rate). Participation in the survey was completely voluntary and anonymous.

### 2.3. Data Collection Tools

The survey was administered in English using Microsoft Forms. It consisted of four sections; the first was about demographic data, followed by a set of questions asking about cigarette smoking based on the Cigarette Dependence Scale (CDS-12) [25]. The third part was intended for E-cigarette (vape) users, and it collected data using the Penn State Electronic Cigarette Dependence Index (PS-ECDI) [26]. The final section focused on the participants’ perceptions of and opinions on cigarettes and E-cigarettes. The full survey questionnaire, including all items and response options, is provided in the Supplementary Materials (Supplementary File S1).

### 2.4. Statistical Analysis

The data was analyzed using both IBM SPSS version 27 (IBM Corp, Armonk, NY, USA) and JASP version 0.19.3, an open-source statistical software developed by the University of Amsterdam, The Netherlands. The level of significance was set at a *p*-value of 0.05. The normality of data was then determined statistically through kurtosis and skewness and verified visually using Q-Q plots. Parametric and non-parametric tests were applied according to data normality. The mean values and standard deviation (SD) were used for normal data, and the median and interquartile range (IQR) were used for non-normal data.

Invalid responses such as the text “vape” for the numerical question regarding the “age of E-cigarette smoking onset” were removed. As a result, two cigarette smokers (2/87; 2.3%) were excluded from all analyses related to the age of cigarette smoking onset for invalid text responses. Similarly, one participant (1/218; 0.5%) was excluded from any analysis involving the age of E-cigarette smoking onset for an invalid text response. Finally, 18 out of 324 nicotine users (5.6%) did not respond to the harm perception questions and were excluded from the corresponding analysis.

### 2.5. Ethics

This study was approved by the university’s ethical committee (IRB number M-F-H-21-August) and was conducted in accordance with the Declaration of Helsinki. Participation was voluntary with informed consent obtained. No incentives were provided for participation, and all data were handled confidentially.

## 3. Results

### 3.1. Demographics

**Figure 1:**
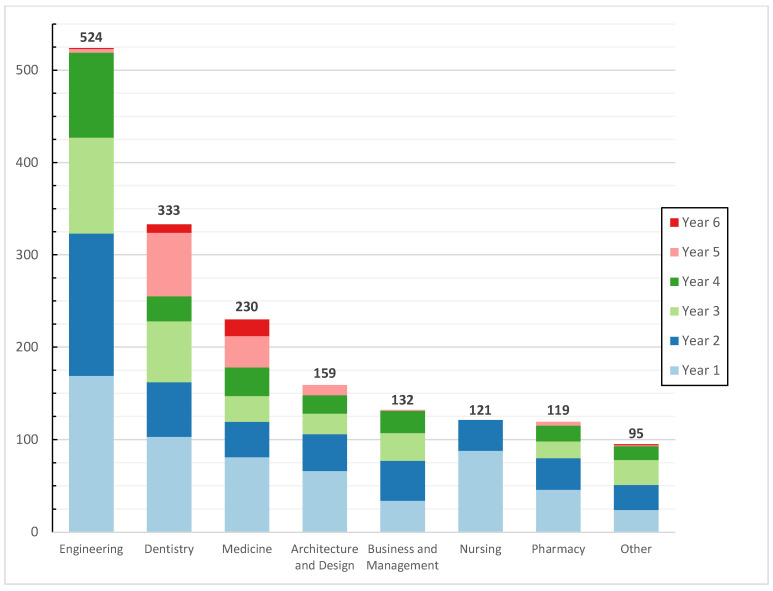
Distribution of responses across different colleges and years.

A total of 1713 students participated in our survey, representing approximately 24% of the total population, of whom 723 were males (42.2%), and 990 were females (57.8%). This gender distribution closely reflects the overall student population, according to the university’s registration department. This is also seen in the college distribution, as shown in Figure 1. The median age of participants was 20.0 years, with an interquartile range (IQR) from 18.0 to 21.0. Most of the responses came from Engineering students, while the least were from College of Law students, with only 10 responses.

As shown in Table 1, non-users of nicotine comprised a larger proportion of responses, reaching 1389 (81.1%), whereas current users accounted for 324 (18.9% of sample) of the total responses. Among these users, their two most used nicotine products were E-cigarettes, 218 (12.7%), and Shisha, 146 (8.5%). On the other hand, cigarettes and pipes were the least smoked, with 87 reporting cigarette use (5.1%) and 15 reporting pipe use (0.9%).

### 3.2. Gender and Nicotine Use

When subdividing nicotine users and non-users based on gender, the female non-user proportion was higher compared to male non-users. Conversely, a larger fraction of current nicotine users comprised males, 226 out of 723 (~31%), in comparison to females, at merely 98 out of 990 (~10%). This is demonstrated in Table 2. A Chi-squared analysis showed a statistically significant difference between gender and nicotine use, with a *p*-value of <0.001. Males had considerably higher odds of being current users than females, with an odds ratio of 4.14 (95% confidence interval: 3.19–5.37).

The mean age of cigarette smoking onset was 16 for males and 16.4 for females, while the mean age of E-cigarette smoking onset was 17 for males and 18 for females. No statistically significant difference was found for the age of onset for cigarette and E-cigarette smoking between males and female, as seen below in Table 3.

### 3.3. Cigarette Dependence Scale Scores

The Cigarette Dependence Scale (CDS) score is calculated from 12 questions completed by participants who currently smoke cigarettes. It ranges from a minimum score of 1 to a maximum of 60, providing a quantitative measure of cigarette dependence, with higher scores indicating greater dependence. In our sample, as shown in Table 4, the mean CDS score was 39.2 ± 11.1, reflecting a moderate to high level of cigarette dependence among traditional cigarette smokers in university. The highest CDS score calculated was 58, while the lowest was 18. There was no significant difference in CDS scores between genders or between participants who had attempted to quit and those who had not. Since no gender differences were observed, the subsequent analyses did not subdivide the sample by gender.

Interestingly, as shown in Table 4, pure traditional cigarette smokers had a significantly lower mean CDS score, 33.6, compared to dual users of both cigarettes and E-cigarettes, who scored higher, 45.4. The mean difference between these groups was 11.8 (95% confidence interval: 6.7–16.9), indicating greater dependence among dual users. Furthermore, a Pearson correlation analysis revealed a significant, weak negative relationship between CDS scores and the age of cigarette smoking onset (r = −0.365, *p* < 0.001), with 13.3% of the variance in dependence explained by the age of initiation (r^2^ = 0.133). Linear regression showed that for every one-year delay in smoking onset, the CDS score decreased by 1.33 points, highlighting the protective effect of delaying smoking initiation against developing severe nicotine dependence.

Finally, the CDS scores of different participants were compared to their age of cigarette smoking onset using Pearson correlation. This revealed a significant weak negative correlation of −0.365 (*p*-value < 0.001) between the two parameters. This yielded an r^2^ of 0.133; thus 13.3% of the variation in CDS scores can be attributed to the age of cigarette smoking onset. Linear regression analysis calculated a slope (M) of −1.33. This predicts a 1.33 decrease in the CDS score with every 1-year increase in cigarette smoking onset age and vice versa.

### 3.4. Penn State Electronic Cigarette Dependence Index

The Penn State Electronic Cigarette Dependence Index (PSECDI) is a 10-item questionnaire that yields a total score ranging from 0 to 20, classifying users into four dependence levels: not dependent (0–3), low dependence (4–8), medium dependence (9–12), and high dependence (13+). Among the 218 E-cigarette users in the sample, 20 (9.2%) were not dependent, 60 (27.5%) had low dependence, 61 (28.0%) had medium dependence, and 77 (35.5%) showed high dependence.

The overall sample demonstrated a medium level of dependence, with a mean PSECDI score of 10.1 (SD = 4.6). Dependence scores did not differ significantly between males and females, aligning with similar findings in cigarette smokers.

As shown in Table 5, students who had previously attempted to quit E-cigarettes reported higher dependence (mean = 10.9, medium dependence) than those who had never attempted to quit (mean = 8.4, low dependence), suggesting that greater dependence may be associated with more attempts to quit smoking, possibly due to failed efforts resulting from stronger nicotine dependence.

A statistically significant difference was also observed between dual users (those who used both E-cigarettes and cigarettes) and exclusive E-cigarette users. Dual users had a higher mean PSECDI score of 12.3, indicating medium dependence, compared to 8.4 among exclusive E-cigarette users, which indicates low dependence. This suggests that dual users may be more strongly dependent on E-cigarettes than those who vape exclusively.

Finally, a statistically significant but weak negative correlation was found between PSECDI scores and the age of onset of E-cigarette use (Pearson’s r = −0.29, *p* < 0.001). In other words, individuals who began vaping at a younger age tended to exhibit higher dependence levels. While modest in strength, this relationship highlights early initiation as a potential risk factor for increased E-cigarette dependence.

**Figure 2:**
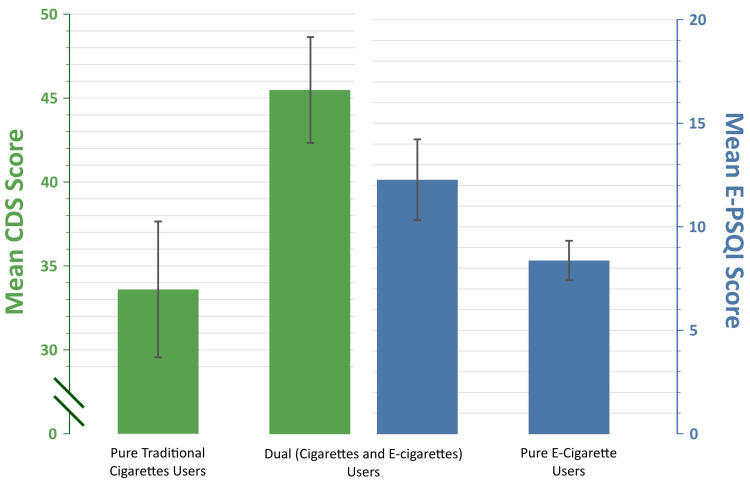
Dependence scores for pure users and dual users with 95% confidence intervals.

Figure 2 highlights both the CDS and E-PSQI scores of dual users of traditional cigarettes and E-cigarettes compared to exclusive users of each product. Dual users of both traditional cigarettes and E-cigarettes showed higher levels of nicotine dependence compared to people who only used one type of product. This may be because using both products leads to higher overall nicotine intake and more frequent opportunities to smoke or vape. Users may alternate between cigarettes and E-cigarettes depending on several factors such as availability and social acceptability. Using both products can create stronger habits, with more triggers or cues that keep reinforcing the behavior. These combined effects result in dual users having an increased dependence on both cigarettes and E-cigarettes, making it more difficult to stop using either product or to cut back.

### 3.5. Harm Perception

As seen in Table 6, participants were asked three questions to assess their perceptions of the health risks associated with cigarette and E-cigarettes use. The first two questions measured participants’ perceptions, asking “how much do you think cigarettes harm health?” and “how much do you think E-cigarettes harm health?”. Responses ranged from 0 to 10, with higher values representing more harm. The last question, “E-cigarettes are as harmful to health as cigarettes”, directly compared the perceived harm of cigarettes and E-cigarettes. Scores from 1 to 10 were obtained, where a score of 1 indicates full disagreement, and 10 indicates full agreement.

Overall, non-nicotine users attributed more harm to both cigarettes and E-cigarettes compared to current users with a statistically significant *p*-value (<0.001). For cigarettes, the median score for harm perception was 10.0, while it was 9.0 for current nicotine users. As for E-cigarettes, the median score for harm for non-users was also 10.0, but current users had a median score of 8.0. The same pattern was observed in the last question comparing cigarettes and E-cigarettes directly with non-nicotine users, who had a median score of 10.0, while current users had a median of 8.0.

The difference in harm perceptions between nicotine users and non-users was greater for E-cigarettes compared to cigarettes. However, it is important to note that both groups recognized the harm caused by E-cigarettes and cigarettes but to different extents.

Participants attributing a higher harm score to cigarettes tended to also do the same for E-cigarettes, with a statistically significant moderate positive correlation between the scoring of both cigarette and E-cigarette harm. Spearman’s rho was 0.615 with a *p*-value of <0.001.

The higher the CDS score, the greater the overall perceived harm of both cigarettes and E-cigarettes and the more agreement that E-cigarettes are as harmful as cigarettes. This weak positive correlation becomes weaker when comparing cigarette harm to E-cigarette harm and finally to the perception that E-cigarettes are as bad as cigarettes. Trending down as such, values of 0.33, 0.30, and 0.23 were obtained. The PS-ECDI score did not have any relation to the perceived harm of both cigarettes and E-cigarettes along with cigarette and E-cigarette use versus the use of both. This is shown in Table 7.

## 4. Discussion

### 4.1. E-Cigarette and Cigarette Use Patterns

As shown in Table 8, data in the region is limited, especially regarding e-cigarettes use among university students. The data shows variability in nicotine product use across UAE universities, with E-cigarettes use ranging from 6.6% to 22.5% and cigarette use ranging from 5.1% to 15.1% [14,27,28]. Even comparisons within the Middle East cannot be made since data, aside from that on university studies, is limited [29]. Further studies in the region are needed to assess the overall trends in nicotine use.

Similar patterns of E-cigarettes use have been reported across other countries, including China (16.7%) and Australia (13.1%), whereas countries such as Turkey report markedly lower use (4.0%) due to a national ban on the sale and import of E-cigarettes. This rising popularity of E-cigarettes among youth is particularly concerning, as exposure to marketing and advertising has been shown to correlate with a higher risk of nicotine initiation and ongoing use [30]. These findings highlight a growing trend of E-cigarettes uptake among university students, often exceeding traditional cigarette use, and underscore the need for targeted educational and regulatory interventions.

**Table 8:** Proportion of smokers found by different studies in the UAE and different regions.

Study and Reference	Population (Sample Size)	E-Cigarettes Prevalence (%)	Cigarette Prevalence (%)
Abbasi et al. (2022) [14]	All UAE * universities (n = 240)	22.5	6.7
Al Sabbah et al. (2022) [27]	Dubai universities (n = 1538)	-	15.7
Ahmed et al. (2021) [28]	UoS *, UAEU *, ZU * (n = 918)	6.6	15.1
Abdulrahman et al. (2022) [31]	Public university students in Riyadh, Saudi Arabia (n = 895)	5.0	18.3
Song et al. (2023) [32]	Students from 7 universities in Guangzhou, China (n = 9361)	16.7	35.0
Ghanim et al. (2024) [33]	5 universities across the West Bank (n = 1002)	19.7	16.0
Dilektaşlı et al. (2024) [34]	Medical university students in Turkey (n = 1054)	4.0	17.0
Kamoni et al. (2025) [35]	University student population in Melbourne, Australia (n = 1094)	13.1	-
Vahratian et al. (2025) [36]	Young adults ages 21–24 in the USA (no n reported)	15.5	-
Pan et al. (2022) [37]	14 countries (no n reported)	0.02 (India) to 3.5 (Russia)	-
Sun et al. (2022) [38]	Youth 12–16 years in 68 countries (n = 485,746)	9.2	-
GDB (2023) [39]	204 countries and territories (no n reported)	-	32.7 men 6.62 women

* UAE, United Arab Emirates; UoS, University of Sharjah; UAEU, United Arab Emirates university; ZU, Zayed university.

In our sample, E-cigarettes use was highly prevalent and accompanied by a high level of dependence, consistent with the findings from other recent university-based studies. When looking at general population studies, the prevalence of E-cigarettes use appears to be concentrated among young adults and in high-income countries [36]. The comparatively low rate of conventional cigarette use in our sample may reflect a shift toward E-cigarettes, which are perceived as more socially acceptable, less regulated, and more appealing to the youth. Flavor variety is a major factor influencing E-cigarettes use, particularly among younger individuals. Studies have shown that users of fruit, mint, and menthol flavors vape more frequently than those using tobacco flavors [40] and that flavor availability remains a key motivator for use [23,24]. As the awareness and accessibility of E-cigarettes continue to expand globally, a further rise in use may be expected, especially among university students and young adults. Implementing targeted educational initiatives and stricter marketing and sales regulations is essential to prevent the normalization of E-cigarettes use and achieve lower use rates, as seen in Turkey’s study [34].

### 4.2. Sex-Based Patterns of Nicotine Use

We found that males were four times more likely to use nicotine products compared to females. This falls in line with the findings from other studies, which generally supported that males in comparison to females tend to have higher nicotine metabolite levels, make up a higher percentage of smokers, and have a higher rate of usage of all tobacco products [41,42]. Cultural beliefs and physiology might play a role in the observed difference. Products like midwakh and shisha are deeply embedded in Arab culture in social gatherings and the like. Smoking in public is widely accepted among men in many Middle Eastern societies but remains socially stigmatized for women. This results in the underreporting of smoking among females and a genuine gender gap in usage patterns. Women who do smoke often do so privately or choose discreet products like E-cigarettes, which have less odor and social visibility. Cultural expectations around modesty and reputation therefore suppress both the reporting and visibility of female nicotine use.

Neuroimaging supported the fact that smoking behavior does differ between females and males on a physiological level. In men, smoking activates the reward system more than in women [43]. In contrast to men, women tend to smoke to regulate mood [44]. This highlights the importance of different sex-specific prevention and cessation strategies, such as focusing on reducing reward-driven reinforcement in men or addressing stress and emotional regulation in women. These targeted campaigns might prove more beneficial than generic broad campaigns.

### 4.3. Preceptions and Early Initiation

Although nicotine users rated the harm of cigarettes and E-cigarettes slightly lower than non-users, both groups acknowledged their health risks. This indicates that many continue to smoke despite an awareness of the dangers, suggesting an acceptance of these risks or perceived benefits such as stress relief. Social influences, addiction, and limited access to cessation resources may also play a role. These findings imply that knowledge alone is insufficient to change behavior, emphasizing the need for youth-centered and accessible cessation programs. The mean age of cigarette initiation at 16 further highlights the urgency of implementing preventive interventions at the secondary school level. Early initiation occurs during a vulnerable neurodevelopmental period when the prefrontal cortex is still maturing; neuroimaging evidence by Miller et al. [45] links early nicotine exposure to structural brain changes, reinforcing the importance of early and targeted prevention strategies.

It is important to note, however, that our correlation coefficients between the age of nicotine onset and dependence level for both cigarettes and E-cigarettes were relatively low (−0.37 and −0.33, respectively). This highlights that although earlier initiation is associated with higher dependence, the relationship is not strong enough to be explained by age alone. Additional factors such as genetic susceptibility, frequency and intensity of use, stress, peer influence, and exposure to advertising may have a larger influence on dependence.

### 4.4. Dependence and Dual Use of E-Cigarettes and Cigarettes

Dependence scores for both traditional cigarettes and E-cigarettes were higher in dual users compared to single users. These results align with studies showing higher dependence in dual users [46], though others reported no difference [43] or even reduced dependence [47]. More nuanced analyses suggest that the frequency of use determines outcomes, with high-frequency dual users being the most dependent and least likely to quit [48]. While E-cigarettes are often promoted as cessation tools, our findings suggest that dual use may instead increase dependence rather than reduce it. This highlights the risk that E-cigarettes, when used alongside conventional cigarettes, may undermine harm reduction goals and complicate quitting efforts. Therefore, public health messages in the region should advise against dual use and frame E-cigarettes not as a safe substitute but as a potential driver of stronger addiction, especially among the youth.

### 4.5. Study Strengths and Limitations

Our study addresses a key gap in the UAE literature by providing updated data on cigarette and E-cigarettes use among university students, a population and region with limited epidemiological evidence. The large sample enhances the representativeness and statistical power of the findings, while validated questionnaires such as the CDS-12 and PS-ECDI ensure the reliable measurement of dependence.

Despite these strengths, several limitations should be acknowledged. This study being limited to Ajman University students means that the findings might not be generalizable for other institutions or among the public. The sampling method used results in reporting and response bias, which could have led to the underreporting of the patterns of nicotine use. The cultural beliefs and stigma surrounding smoking may have even resulted in the difference in patterns seen among genders. Although validated tools were used to quantify dependence for both cigarette and E-cigarettes use, recall bias still exists. In addition, questions about the perception of nicotine smoking can be very subjective. Not to mention, the two scales used were applied independently for dual users, therefore affecting the accuracy of assessing dual users. In retrospect, further questions regarding shisha use were required, as we did not expect such a significant pattern for shisha use.

## 5. Conclusions

Despite the widespread awareness of the risks of cigarettes and E-cigarettes among university students, nicotine use remains prevalent among them, with 18.9% of participants being nicotine users. More than half, 12.1%, of nicotine users used E-cigarettes. Furthermore, dependence on both E-cigarettes and cigarettes was moderately high in Ajman University’s student population. Being male was identified as a risk factor for nicotine use, while dual nicotine use and an earlier age of nicotine initiation were associated with more nicotine dependence in our sample. Although E-cigarettes are often promoted as smoking cessation tools, their use in our sample did not appear to serve this purpose, suggesting that they may instead sustain nicotine dependence. This further highlight nicotine use, especially E-cigarettes use among the youth, as a pressing issue that requires a different approach, such as focusing more on targeted rehabilitation programs, along with stricter e-cigarette policies and marketing. Schools and universities should focus on delaying initiation through early prevention and first-year interventions that emphasize behavioral skills (e.g., refusal and stress-coping), supported by comprehensive smoke- and vape-free campus policies. Further longitudinal studies are required to assess the trend in e-cigarette use and its possible effect on health.

## Figures and Tables

**Table 1:** Data demographics.

Variables	*N* (%)	Median (IQR)
Gender		
Male	723 (42.2)	-
Female	990 (57.8)	-
Nicotine Status		
Current user	324 (18.9)	-
Non-user	1389 (81.1)	-
Nicotine Products Used ^1^		
E-cigarettes (vape)	218 (12.7)	-
Shish	146 (8.5)	-
Midwakh	97 (5.7)	-
Cigarettes	87 (5.1)	-
Pipe	15 (0.9)	-
Age	-	20.0 (18–21)

^1^ Participants were allowed to select more than one option; as a result, totals exceed the number of smokers.

**Table 2:** Contingency table of gender and nicotine use.

Nicotine Use	Gender		Total (%)
	Female (%)	Male (%)	
Non-user	892 (52.1)	497 (29.0)	1389 (81.1)
Current user	98 (5.7)	226 (13.2)	324 (18.9)
Total	990 (57.8)	723 (42.2)	1713 (100)

Chi-squared test value is 124.3, *p*-value < 0.001.

**Table 3:** Onset age of nicotine use between genders.

Variables	Categories	Mean ± SD	t/U	*p*-Value
Age of cigarette smoking onset	Male	16 ± 3.2		
	Female	16.4 ± 2.7	0.349	0.728
Age of E-cigarette smoking onset	Male	17.0 (15–18) *		
	Female	18.0 (16–19) *	5337.5	0.097

* Values reflect the median and (interquartile range).

**Table 4:** An overview of the Cigarette Dependence Scale (CDS) score results across different groups.

	*N* (%)	Mean ± SD	95% CI	t	*p*-Value
Total	87 (100)	39.2 ± 11.1			
Male	73 (83.9)	38.6 ± 11.5	36.0 to 41.1		
Female	14 (16.1)	42.7 ± 11.0	36.1 to 49.4	1.276	0.205
Cigarette Smoking Cessation					
Attempted to quit	58 (66.7)	37.0 ± 11.3	37.5 to 43.2		
Did not attempt to quit	29 (33.3)	40.4 ± 10.9	32.7 to 41.3	−1.322	0.190
Cigarette use group					
Pure cigarettes	22	33.6 ± 9.8	29.3 to 37.9		
Dual cigarettes and E-cigarettes	28	45.4 ± 8.2	42.2 to 48.6	−2.797	<0.001

**Table 5:** An overview of the Penn State Electronic Cigarette Dependence Index (PSECDI) score results.

	*N* (%)	Mean ± SD	95% CI	t	*p*-Value
Total	218 (100)	10.1 ± 4.6			
Male	158 (72.5)	10.3 ± 4.5	9.6 to 11.0		
Female	60 (27.5)	9.5 ± 4.7	9.5 to 10.7	1.255	0.21
E-cigarettes cessation					
Attempted to quit	144 (66.1%)	10.9 ± 4.4	10.2 to 11.6		
Did not attempt to quit	74 (33.9%)	8.4 ± 4.6	7.4 to 9.5	−3.893	<0.001
E-cigarettes use group					
Pure E-cigarettes	63	8.4 ± 5.1	7.4 to 9.4		
Dual E-cigarettes and cigarettes	28	12.3 ± 4.1	10.3 to 14.3	3.909	<0.001

**Table 6:** Perceptions of harm between nicotine users and non-nicotine users.

	Median Score (IQR)	U	*p*-Value
Cigarettes harm health			
Non-nicotine users	10.0 (9.0–10.0)		
Current nicotine users	9.0 (7.0–10.0)	266,039.5	>0.001
E-cigarettes (Vapes) harm health			
Non-nicotine users	10.0 (8.0–10.0)		
Current nicotine users	8.0 (7.0–10.0)	265,281.5	>0.001
E-cigarettes are as bad as ordinary cigarettes			
Non-nicotine users	10.0 (8.0–10.0)		
Current nicotine users	8.0 (5.0–10.0)	262,752.5	>0.001

**Table 7:** Correlation between dependence scores and harm perceptions.

Independent Variable	Dependent Variables	Spearman Rho	*p*-Value
Extent that cigarettes harm health			
	Extent that E-cigarettes (Vapes) harm health	0.615	<0.001
Cigarette Dependence Scale (CDS) score			
	Extent that cigarettes harm health	0.330	0.003
	Extent that E-cigarettes (vapes) harm health	0.302	0.007
	E-cigarettes are as bad as ordinary cigarettes	0.233	0.037
Penn State E-cigarette Dependence Index (PS-ECDI) score			
	Cigarettes harm health	0.069	0.320
	E-cigarettes (vapes) harm health	0.073	0.297
	E-cigarettes are as bad as ordinary cigarettes	0.009	0.893

## Data Availability

The dataset is available on request from the authors.

## Supplementary Materials

The following supporting information can be downloaded at https://www.mdpi.com/article/10.3390/ijerph23020143/s1, File S1: Survey questionnaire (Microsoft Forms).

## References

[1] Castaldelli-Maia J.M., Ventriglio A., Bhugra D. (2016). Tobacco Smoking: From ‘Glamour’ to ‘Stigma’. A Comprehensive Review. Psychiatry Clin. Neurosci..

[2] Almogbel F., Almuqbil S., Rabbani U., Almogbel Y. (2020). Prevalence and Predictors of Midwakh Smoking among Male Students of Qassim University, Al-Qassim, Saudi Arabia. Tob. Induc. Dis..

[3] SDG Target 3.a Tobacco Control. https://www.who.int/data/gho/data/themes/topics/sdg-target-3_a-tobacco-control.

[4] Nasser A.M.A., Geng Y., Al-Wesabi S.A. (2020). The Prevalence of Smoking (Cigarette and Waterpipe) among University Students in Some Arab Countries: A Systematic Review. Asian Pac. J. Cancer Prev..

[5] Razzak H.A., Harbi A., Ahli S. (2020). Tobacco Smoking Prevalence, Health Risk, and Cessation in the UAE. Oman Med. J..

[6] Bartal M. (2001). Health Effects of Tobacco Use and Exposure. Monaldi Arch. Chest Dis..

[7] CDC Health Effects of Cigarettes: Cardiovascular Disease. https://www.cdc.gov/tobacco/about/cigarettes-and-cardiovascular-disease.html.

[8] Centers for Disease Control and Prevention (US), National Center for Chronic Disease Prevention and Health Promotion (US), Office on Smoking and Health (US) (2010). How Tobacco Smoke Causes Disease: The Biology and Behavioral Basis for Smoking-Attributable Disease: A Report of the Surgeon General.

[9] CDC Health Effects of Cigarettes: Cancer. https://www.cdc.gov/tobacco/about/cigarettes-and-cancer.html.

[10] E-cigarettes: Facts, Stats and Regulations. https://truthinitiative.org/research-resources/emerging-tobacco-products/E-cigarettes-facts-stats-and-regulations.

[11] Overbeek D.L., Kass A.P., Chiel L.E., Boyer E.W., Casey A.M. (2020). A Review of Toxic Effects of Electronic Cigarettes/Vaping in Adolescents and Young Adults. Crit. Rev. Toxicol..

[12] Salari N., Rahimi S., Darvishi N., Abdolmaleki A., Mohammadi M. (2024). The Global Prevalence of E-cigarettes in Youth: A Comprehensive Systematic Review and Meta-Analysis. Public Health Pract..

[13] Jirjees F., Bashi Y.H.D., Kharaba Z., Ahmadi K., Barakat M., AlObaidi H. (2023). Public Awareness, Prevalence, and Regulations for the Sale of Electronic Cigarettes in Arab Countries: A Narrative Review. Tob. Induc. Dis..

[14] Abbasi Y., Hout M.-C.V., Faragalla M., Itani L. (2022). Knowledge and Use of Electronic Cigarettes in Young Adults in the United Arab Emirates, Particularly during the COVID-19 Pandemic. Int. J. Environ. Res. Public Health.

[15] Wagener T.L., Avery J.A., Leavens E.L.S., Simmons W.K. (2021). Associated Changes in E-Cigarette Puff Duration and Cigarettes Smoked per Day. Nicotine Tob. Res..

[16] Hanewinkel R. (2023). Electronic cigarettes: Harm reduction or harm prolongation?. Pneumologie.

[17] Ran S., Yang J.J., Piper M.E., Lin H.-C., Buu A. (2024). Health Risks Associated with Adopting New-Generation Disposable Products Among Young Adults Who Use E-cigarettes. Int. J. Environ. Res. Public Health.

[18] Ou T.-S., Buu A., Yang J.J., Lin H.-C. (2024). E-Cigarette Use Reasons and Associated e-Cigarette Use Dependence among College Students: A Longitudinal Examination. Addict. Behav..

[19] Yang Q., Clendennen S.L., Marti C.N., Loukas A. (2024). Associations between Social Media Engagement and Young Adults’ Subsequent Onset of ENDS Dependence Symptoms One Year Later. Addict. Behav..

[20] Henn S.L., Martinasek M.P., Lange M. (2023). Vaping Behavior in Young Adults During the COVID-19 Pandemic. Respir. Care.

[21] E-Cigarette or Vaping Product Use-Associated Lung Injury (EVALI)—UpToDate. https://www.uptodate.com/contents/e-cigarette-or-vaping-product-use-associated-lung-injury-evali/print.

[22] Zulfiqar H., Sankari A., Rahman O. (2025). Vaping-Associated Pulmonary Injury. StatPearls.

[23] American Lung Association E-Cigarette or Vaping Use-Associated Lung Injury (EVALI). https://www.lung.org/lung-health-diseases/lung-disease-lookup/evali.

[24] QS Top Universities—Ajman University. https://www.topuniversities.com/universities/ajman-university.

[25] Etter J.-F., Le Houezec J., Perneger T.V. (2003). A Self-Administered Questionnaire to Measure Dependence on Cigarettes: The Cigarette Dependence Scale. Neuropsychopharmacol.

[26] Foulds J., Veldheer S., Yingst J., Hrabovsky S., Wilson S.J., Nichols T.T., Eissenberg T. (2015). Development of a Questionnaire for Assessing Dependence on Electronic Cigarettes Among a Large Sample of Ex-Smoking E-Cigarette Users. Nicotine Tob. Res..

[27] Al Sabbah H., Assaf E.A., Dabeet E. (2022). Prevalence of Smoking (Cigarette and Waterpipe) and Its Association with Obesity/Overweight in UAE and Palestine. Front. Public Health.

[28] Ahmed L.A., Verlinden M., Alobeidli M.A., Alahbabi R.H., AlKatheeri R., Saddik B., Oulhaj A., Al-Rifai R.H. (2021). Patterns of Tobacco Smoking and Nicotine Vaping among University Students in the United Arab Emirates: A Cross-Sectional Study. Int. J. Environ. Res. Public Health.

[29] Al-Hamdani M., Brett Hopkins D. (2023). E-cigarettes in the Middle East: The Known, Unknown, and What Needs to Be Known Next. Prev. Med. Rep..

[30] Hansen J., Hanewinkel R., Morgenstern M. (2020). Electronic Cigarette Advertising and Teen Smoking Initiation. Addict. Behav..

[31] Bin Abdulrahman K.A., Alghamdi H.A., Alfaleh R.S., Albishri W.S., Almuslamani W.B., Alshakrah A.M., Alsuwailem H.M., Alkhelaiwi S.A. (2022). Smoking Habits among College Students at a Public University in Riyadh, Saudi Arabia. Int. J. Environ. Res. Public Health.

[32] Song H., Yang X., Yang W., Dai Y., Duan K., Jiang X., Huang G., Li M., Zhong G., Liu P. (2023). Cigarettes Smoking and E-cigarettes Using among University Students: A Cross-Section Survey in Guangzhou, China, 2021. BMC Public Health.

[33] Ghanim M., Rabayaa M., Abuawad M., Saeedi M., Amer J. (2024). E-Cigarette Use among University Students in Palestine: Prevalence, Knowledge, and Determinant Factors. PLoS ONE.

[34] Dilektasli A.G., Guclu O.A., Ozpehlivan A., Durak V.A., Gezmis I., Ozgur A., Cinar B., Demirdogen E., Ozturk N.A.A., Ozkaya G. (2024). Electronic Cigarette Use and Consumption Patterns in Medical University Students. Front. Public Health.

[35] Kamoni T., Selamoglu M., Osadnik C., Madawala S., Kotwas S., Turudia K., Barton C. (2025). E-Cigarette Use and Health Information Needs among a University Student Population in Melbourne, Australia. Front. Public Health.

[36] Vahratian A., Briones E.M., Jamal A., Marynak K.L. (2025). Electronic Cigarette Use Among Adults in the United States, 2019–2023. NCHS Data Briefs.

[37] Pan L., Morton J., Mbulo L., Dean A., Ahluwalia I.B. (2022). Electronic Cigarette Use among Adults in 14 Countries: A Cross-Sectional Study. EClinicalMedicine.

[38] Sun J., Xi B., Ma C., Zhao M., Bovet P. (2022). Prevalence of E-Cigarette Use and Its Associated Factors Among Youths Aged 12 to 16 Years in 68 Countries and Territories: Global Youth Tobacco Survey, 2012–2019. Am. J. Public Health.

[39] GBD 2021 Diseases and Injuries Collaborators (2024). Global Incidence, Prevalence, Years Lived with Disability (YLDs), Disability-Adjusted Life-Years (DALYs), and Healthy Life Expectancy (HALE) for 371 Diseases and Injuries in 204 Countries and Territories and 811 Subnational Locations, 1990-2021: A Systematic Analysis for the Global Burden of Disease Study 2021. Lancet.

[40] Do E.K., O’Connor K., Kreslake J.M., Friedrichsen S.C., Vallone D.M., Hair E.C. (2022). Influence of Flavors and Nicotine Concentration on Nicotine Dependence in Adolescent and Young Adult E-Cigarette Users. Subst. Use Misuse.

[41] Chen A., Krebs N.M., Zhu J., Sun D., Stennett A., Muscat J.E. (2017). Sex/Gender Differences in Cotinine Levels Among Daily Smokers in the Pennsylvania Adult Smoking Study. J. Women’s Health.

[42] Chinwong D., Mookmanee N., Chongpornchai J., Chinwong S. (2018). A Comparison of Gender Differences in Smoking Behaviors, Intention to Quit, and Nicotine Dependence among Thai University Students. J. Addict..

[43] Cosgrove K.P., Wang S., Kim S.-J., McGovern E., Nabulsi N., Gao H., Labaree D., Tagare H.D., Sullivan J.M., Morris E.D. (2014). Sex Differences in the Brain’s Dopamine Signature of Cigarette Smoking. J. Neurosci..

[44] Sieminska A., Jassem E. (2014). The Many Faces of Tobacco Use among Women. Med. Sci. Monit..

[45] Miller A.P., Baranger D.A.A., Paul S.E., Garavan H., Mackey S., Tapert S.F., LeBlanc K.H., Agrawal A., Bogdan R. (2024). Neuroanatomical Variability and Substance Use Initiation in Late Childhood and Early Adolescence. JAMA Netw. Open.

[46] Martínez Ú., Martínez-Loredo V., Simmons V.N., Meltzer L.R., Drobes D.J., Brandon K.O., Palmer A.M., Eissenberg T., Bullen C.R., Harrell P.T. (2019). How Does Smoking and Nicotine Dependence Change After Onset of Vaping? A Retrospective Analysis of Dual Users. Nicotine Tob. Res..

[47] Kaplan B., Alrumaih F., Breland A., Eissenberg T., Cohen J.E. (2020). A Comparison of Product Dependence among Cigarette Only, ENDS Only, and Dual Users: Findings from Wave 3 (2015–2016) of the PATH Study. Drug Alcohol Depend..

[48] Azagba S., Shan L., Latham K. (2019). Adolescent Dual Use Classification and Its Association With Nicotine Dependence and Quit Intentions. J. Adolesc. Health.

