# Supplementary material for: Traditional and Electronic Cigarette Usage Patterns, Dependence, and Perceptions Among Ajman University Students
Source: Int J Environ Res Public Health. 2026 Jan 23;23(2):143. doi: 10.3390/ijerph23020143 (PMC12941306; doi:10.3390/ijerph23020143)
Supplement: Supplementary file 1 [file ijerph-23-00143-s001.zip › ijerph-4039380-SI.pdf]

SUPPLEMENTARY FILE S1: SURVEY QUESTIONNAIRE (MICROSOFT FORMS)

DEMOGRAPHICS

1.AGE
"Free text"

2.SEX
Male
Female

3.UNIVERSITY ID
"Free text"

4.ACADEMIC PROGRAM
Bachelor of Medicine and Bachelor of Surgery/Doctor of Medicine
Bachelor of Dental Surgery
Bachelor of Pharmacy (BPharm)
Bachelor of Science in Engineering
Bachelor of Architecture/Bachelor of Interior Design
Bachelor of Science in Managment/Marketing/Accounting
Bachelor of Arts in Sociology and Social Work
Bachelor of Mass Communication
Bachelor of Law
Bachelor of Science in Nursing
Other

5.YEAR OF STUDY
1
2
3
4
5
6

CDS

6.DO YOU INHALE ANY NICOTINE PRODUCTS? (CIGARETTES, VAPES, SHISHA, ETC...)
Yes
No

7.DO YOU SMOKE CIGARETTES CURRENTLY?
Yes
No

8.AT WHAT AGE DID YOU START SMOKING?
“Free text”

9.PLEASE RATE YOUR ADDICTION TO CIGARETTES
“Free text”

10.ON AVERAGE, HOW MANY CIGARETTES DO YOU SMOKE PER DAY?
0-5 Cigarettes / day
6-10 Cigarettes / day
11-20 Cigarettes / day
21-29 Cigarettes / day
30+ Cigarettes / day

11.USUALLY, HOW SOON AFTER WAKING UP DO YOU SMOKE YOUR FIRST CIGARETTE?
0-5 min
6-15 min
16-30 min
31-60 min
61+ min

12.DO YOU SOMETIMES AWAKEN AT NIGHT TO SMOKE A CIGARETTE?
Yes
No

13.HOW MANY NIGHTS PER WEEK DO YOU TYPICALLY AWAKEN TO SMOKE A CIGARETTE?
0-1 night
2-3 nights
4+ nights

14.FOR YOU, QUITTING SMOKING FOR GOOD WOULD BE:
Impossible
Very difficult
Fairly difficult
Fairly Easy
Very Easy

15.DID YOU TRY QUITTING SMOKING?
Yes
No

16.PLEASE INDICATE WHETHER YOU AGREE WITH EACH OF THE FOLLOWING STATEMENTS:
Totally Disagree
Somewhat Disagree
Neutral
Somewhat Agree
Fully Agree

After a few hours without smoking, I feel an irresistible urge to smoke
The idea of not having any cigarettes causes me stress
Before going out, I always make sure that I have cigarettes with me

I am a prisoner of cigarettes
I smoke too much
Sometimes I drop everything to go out and buy cigarettes
I smoke all the time
I smoke despite the risks to my health

E-PSQI

17.DO YOU CURRENTLY VAPE OR USE ECIGS

Yes
No

18.AT WHAT AGE DID YOU START SMOKING?

“Free text”

19.HOW MANY TIMES PER DAY DO YOU USUALLY USE YOUR ELECTRONIC CIGARETTE? (ASSUME THAT ONE “TIME” CONSISTS OF AROUND 15 PUFFS OR LASTS AROUND 10 MINUTES)

0 - 4 times/day
5 - 9
10 - 14
15 - 19
20 - 29
30+

20.ON DAYS THAT YOU CAN USE YOUR ELECTRONIC CIGARETTE FREELY, HOW SOON AFTER YOU WAKE UP DO YOU FIRST USE YOUR ELECTRONIC CIGARETTE?

0 - 5 mins
6 - 15
16 - 30
31 - 60
61 - 120
121+

21.DO YOU SOMETIMES AWAKEN AT NIGHT TO USE YOUR ELECTRONIC CIGARETTE?

Yes
No

22.HOW MANY NIGHTS PER WEEK DO YOU TYPICALLY AWAKEN TO USE YOUR ELECTRONIC CIGARETTE?

0-1 night
2-3 nights
4+ nights

23.DO YOU USE AN ELECTRONIC CIGARETTE NOW BECAUSE IT IS REALLY HARD TO QUIT (ELECTRONIC CIGARETTES)?

Yes
No

24.DO YOU EVER HAVE STRONG CRAVINGS TO USE AN ELECTRONIC CIGARETTE?
Yes
No

25.OVER THE PAST WEEK, HOW STRONG HAVE THE URGES TO USE AN ELECTRONIC CIGARETTE BEEN?
None/Slight
Moderate/Strong
Very strong/Extremely strong

26.IS IT HARD TO KEEP FROM USING AN ELECTRONIC CIGARETTE IN PLACES WHERE YOU ARE NOT SUPPOSED TO?
Yes
No

27.WHEN YOU HAVEN'T USED AN ELECTRONIC CIGARETTE FOR A WHILE OR WHEN YOU TRIED TO STOP USING…
Did you feel more irritable because you couldn't use an electronic cigarette? (Yes No)
Did you feel nervous, restless, or anxious because you couldn't use an electronic cigarette? (Yes No)

28.FOR YOU, QUITTING VAPING FOR GOOD WOULD BE:
Impossible
Very difficult
Fairly difficult
Fairly Easy
Very Easy

29.DID YOU TRY TO QUIT VAPING?
Yes
No

OTHER NICOTINE PRODUCTS

30.HAVE YOU USED ANY OTHER INHALED NICOTINE PRODUCT NOT MENTIONED ABOVE? (E.G.: SHISHA, PIPE, MEDWAKH,.....)
Yes
No

31.WHICH NICOTINE PRODUCT(S) DID YOU USE?
Shisha
Midwakh
Pipe
Other

32.LIST THE INHALED NICOTINE PRODUCT/S AND HOW FREQUENTLY EACH IS USED.
"free text"

ATTITUDES TOWARD SMOKING AND VAPING

33. DO YOU AGREE THAT SMOKING IS BAD FOR YOUR HEALTH?

Yes
No

34. HOW MUCH DO YOU THINK CIGARETTES HARM HEALTH?

On scale of 0-10 (0 no harm, 10 major significant harm)

35. HOW MUCH DO YOU THINK E-CIGS (VAPES) HARM HEALTH?

On scale of 0-10 (0 no harm, 10 major significant harm)

36. TO WHAT EXTENT DO YOU AGREE/DISAGREE WITH THE FOLLOWING STATEMENT: CIGS ARE AS BAD AS ORDINARY CIGARETTES

On a scale of 1-10, 1 fully disagree, 5 neutral and 10 fully agree
